# Peculiarities of the anhedonia phenomenon in schizophrenic and affective disorders

**DOI:** 10.1192/j.eurpsy.2023.447

**Published:** 2023-07-19

**Authors:** A. Barkhatova, M. Popov

**Affiliations:** Department of endogenous mental disorders and affective states, Mental health research center, Moscow, Russian Federation

## Abstract

**Introduction:**

Anhedonia is a transdiagnostic psychopathological phenomenon, which is assessed as «core» for several diseases, first of all - schizophrenic and affective spectrum disorders. The problem of clinical features differentiation and identification of anhedonia’s neurobiological mechanisms in the structure of the affective and schizophrenic spectrum disorders is still topical and far from being resolved.

**Objectives:**

The aim of the study was comparative research of the relationship between the features of neurocognitive functioning and the manifestations of anhedonia among patients with disorders of the schizophrenic and affective spectra.

**Methods:**

The sample consisted of 40 patients, 17 patients with schizophrenic spectrum disorders and 23 patients with affective spectrum disorders were examined. We used next psychometric techniques to research anhedonia: Revised Social Anhedonia Scale (RSAS), The Temporal Experience of Pleasure Scale (TEPS), Physical Anhedonia Scale (PAS). We used following methods to study neuropsychological features: Dynamic praxis; Color interference test; Arithmetic Tasks; Plot picture; Number of skips and impulsive errors; Reverse and straight rows; Verbal fluency; Number of repetitions; Rey-Osterritz figure.

**Results:**

Patients with schizophrenia spectrum disorders show lower scores of pleasure anticipation ability and ability to experience pleasure in the social sphere. Neuropsychological indicators of prefrontal cortex dysfunction demonstrate a positive relationship (p = 0.035) with the severity of social anhedonia and a negative relationship with the ability to anticipate pleasure. Thalamus and forehead dysfunction indicators mainly show a negative relationship with the ability to directly experience pleasure. Indicators of dysfunction of the parietal, occipital and temporal lobes have single connections with different parameters of anhedonia.

**Conclusions:**

Manifestations of different parameters of anhedonia demonstrate their heterogeneity among patients with schizophrenic and affective spectrum disorders. Patients with schizophrenic spectrum disorders have greater difficulty with anticipation of pleasure and the ability to experience pleasure in the social sphere. The ability to anticipate pleasure is more strongly related to prefrontal brain function, whereas the ability to experience pleasure directly is related to subcortical brain function.

**Disclosure of Interest:**

None Declared

